# Supplementary material for: Size uniformity of animal cells is actively maintained by a p38 MAPK-dependent regulation of G1-length
Source: eLife. 2018 Mar 29;7:e26947. doi: 10.7554/eLife.26947 (PMC5876018; doi:10.7554/eLife.26947)
Supplement: Figure 4—source data 1. — Kd values in the table were extracted from Davis et al. (2011). As marked in that paper, blank fields indicate combinations that were tested, but for which binding was weak (Kd >10 μM), or not detected in a 10 μM primary screen. [file elife-26947-fig4-data1.docx]

**Figure 4 -source data 1**. Binding activity (Kd’s in nM) against each of the p38 isoforms

| **Kinase** | **BIRB-796** | **SB-203580** |
| --- | --- | --- |
| **p38-alpha** | 0.45 | 12 |
| **p38-beta** | 7.2 | 70 |
| **p38-gamma** | 2.9 | 1500 |
| **p38-delta** | 78 |  |

Kd values in the table were extracted from (Davis et al., 2011). As marked in that paper, blank fields indicate combinations that were tested, but for which binding was weak (Kd > 10 uM), or not detected in a 10 uM primary screen.
